# Therapeutic Potential of Cathelicidin Peptide LL-37, an Antimicrobial Agent, in a Murine Sepsis Model

**DOI:** 10.3390/ijms21175973

**Published:** 2020-08-19

**Authors:** Isao Nagaoka, Hiroshi Tamura, Johannes Reich

**Affiliations:** 1 Department of Host Defense and Biochemical Research, Juntendo University Graduate School of Medicine, 2-1-1 Hongo, Bunkyo-ku, Tokyo 113-8421, Japan; 2 Department of Physical Therapy, Faculty of Health Science, Juntendo University, 3-2-12 Hongo, Bunkyo-ku, Tokyo 113-0033, Japan; 3 Laboratory Program Support (LPS) Consulting Office, 4-7-13 Nishi-Shinjuku, Shinjuku-ku, Tokyo 160-0023, Japan; htamura@lpsct.com; 4 Microcoat Biotechnologie GmbH, Am Neuland 3, 82347 Bernried am Starnberger See, Germany; j.reich@microcoat.de

**Keywords:** antimicrobial peptide, cathelicidin, sepsis, pyroptosis, NETs, ectosome

## Abstract

Among the mechanisms put-up by the host to defend against invading microorganisms, antimicrobial peptides represent the first line. In different species of mammals, the cathelicidin family of antimicrobial peptides AMPs has been identified, and in humans, LL-37 is the only type of cathelicidin identified. LL-37 has many different biological activities, such as regulation of responses to inflammation, besides its lipopolysaccharide (LPS)-neutralizing and antimicrobial and activities. Recently, employing a murine septic model that involves cecal ligation and puncture (CLP), we examined the effect of LL-37. The results indicated that LL-37 exhibits multiple protective actions on septic mice; firstly, the survival of CLP mice was found to be improved by LL-37 by the suppression of the macrophage pyroptosis that induces the release of pro-inflammatory cytokines (such as IL-1β) and augments inflammatory reactions in sepsis; secondly, the release of neutrophil extracellular traps (NETs), which have potent bactericidal activity, is enhanced by LL-37, and protects mice from CLP-induced sepsis; thirdly, LL-37 stimulates neutrophils to release antimicrobial microvesicles (ectosomes), which improve the pathological condition of sepsis. These findings indicate that LL-37 protects CLP septic mice through at least three mechanisms, i.e., the suppression of pro-inflammatory macrophage pyroptosis and the release of antimicrobial NETs (induction of NETosis) and ectosomes from neutrophils. Thus, LL-37 can be a potential therapeutic candidate for sepsis due to its multiple properties, including the modulation of cell death (pyroptosis and NETosis) and the release of antimicrobial NETs and ectosomes as well as its own bactericidal and LPS-neutralizing activities.

## 1. Introduction

Sepsis is a frequent cause of mortality in the noncoronary intensive care unit (ICU). Sepsis results from harmful or detrimental host response to infection and it is a systemic host response [1,2]. Multiple organ failure, which mostly results from the excessive production of pro-inflammatory cytokines, arises because of the dysregulated inflammatory/immune responses during sepsis. Recent statistics indicate a decrease in sepsis mortality in hospitals, probably due to advances in patient care. Unfortunately, clinical trials revealed that many of the therapeutic approaches with anti-inflammatory cytokines are not effective [2,3]. Therefore, it is necessary to develop an effective and novel approach for sepsis treatment [2,3]. Many recent studies addressed the host cell death mechanisms, which likely underlie the dysregulated inflammatory/immune responses in sepsis [4,5,6].

Macrophages and dendritic cells, which are found in bacterial infection, undergo caspase-1-dependent cell death, called pyroptosis [7]. The cells undergoing pyroptosis release pro-inflammatory cytokines, such as interleukin (IL)-1β and IL-18, extracellularly [7,8]. IL-1β not only enhances both systemic and local inflammatory/immune responses [9] but also leads to tissue injury in sepsis, by synergistically acting along with other cytokines [10].

Neutrophils are the most abundant leukocytes and as an important part of the innate immune system in humans, protect the host against invading microorganisms [11,12]. Upon stimulation, neutrophils go through NETosis (a kind of programmed cell death) and trigger the release of neutrophil extracellular traps (NETs) [13]. NETs are capable of trapping microorganisms and exerting anti-bacterial activity through the NET-associated components’ action (e.g., DNA, histone and granule-derived proteins and peptides) [14,15]. Furthermore, stimulated neutrophils extracellularly release microvesicles (0.1-1.0 μm in diameter), called ectosomes, during the inflammatory response [16,17]. Ectosomes contain functional proteins of neutrophils [17,18], and exhibit bacteriostatic potential [19]. Notably, the ectosome level is reported to be augmented in surviving patients of sepsis [20,21].

**Figure 1:**
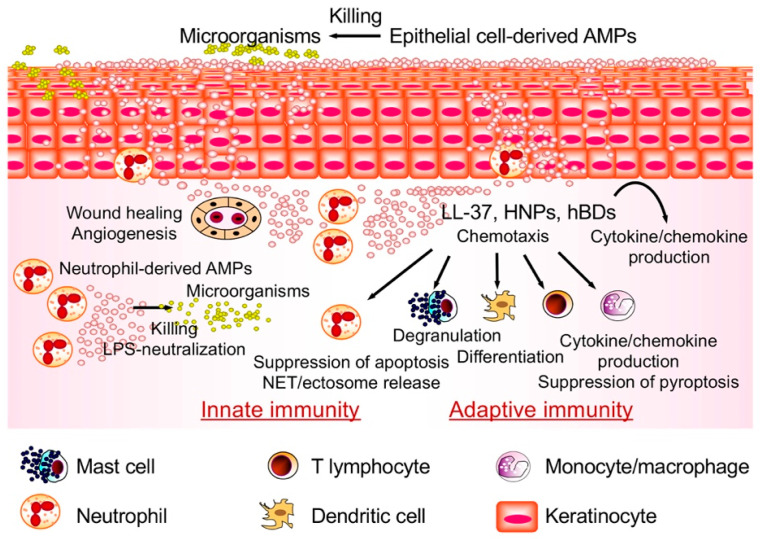
Roles of antimicrobial peptides in host defense. Antimicrobial peptides (AMPs) participate in the innate immune response by showing antimicrobial actions against Gram-negative and -positive bacteria, viruses and fungi. Besides having direct antimicrobial properties, AMPs also have the capability to augment immunity and to link adaptive and innate immunity by acting on host cells as immunomodulators. HNPs, human neutrophil peptides; hBDs, human β-defensins.

Antimicrobial peptides are evolutionarily conserved among various species (vertebrates as well as invertebrates, the latter encompassing arthropods) [22,23]. These are also known as host defense peptides and take part in the innate immune response by exhibiting antimicrobial activities against both Gram-negative and -positive bacteria, and also viruses and fungi. Antimicrobial peptides (AMPs) belong to two main families, the defensins and cathelicidins. Besides acting as antimicrobials, AMPs are also capable of enhancing immunity by acting on host cells as immunomodulators to link innate and adaptive immunity [22,23] (Figure 1).

Nearly 30 different cathelicidin types have been described in various species of mammals. In humans, however, there is only one type of cathelicidin, known as the human cationic antibacterial protein of 18 kDa (hCAP18). The hCAP18 has a C-terminal mature antibacterial peptide LL-37, which consists of 37 amino acid residues with the first two leucine residues (L^1^LGDFFRKSKEKIGKEFKRIVQRIKDFLRNLVPRTES^37^) and has been found mostly in epithelial cells and neutrophils [24,25,26]. Besides having antimicrobial and lipopolysaccharide (LPS)-neutralizing activities [27,28], LL-37 also shows many biological activities, such as regulation of responses to inflammation [26,29]. Importantly, we demonstrated before that in neutrophils the spontaneous apoptosis is inhibited by LL-37 through purinergic receptor P2X_7_ as well as formyl-peptide receptor-like 1 (FPRL1) [30]. We also showed that LL-37, by neutralizing the action of LPS, decreases the apoptosis of endothelial cells induced by LPS [31]. These above results suggested that cell death is modulated by LL-37. Recently, we assessed the influence of LL-37 in a murine cecal ligation and puncture (CLP) sepsis model and revealed that the survival of CLP septic mice is improved by the administration of LL-37, which also caused the suppression of macrophage pyroptosis [32] and the release of NETs [33] and ectosomes [34] from neutrophils. Thus, in this article, based on our recent findings, the therapeutic potential of LL-37 on a murine sepsis model is reviewed.

## 2. Inhibition of the Macrophage Pyroptosis and Improvement of the CLP Mouse Survival by LL-37

**Figure 2:**
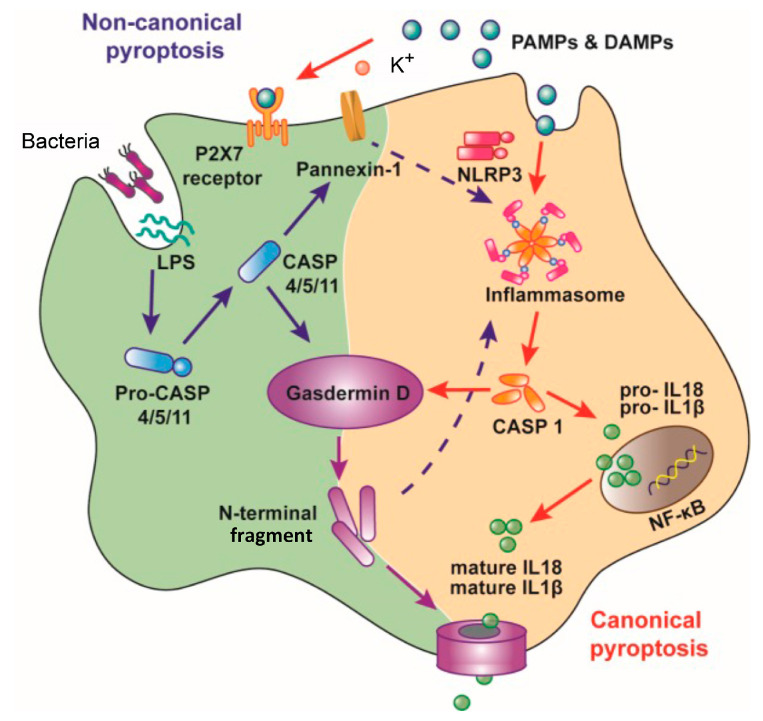
Mechanism for the induction of pyroptosis. Pyroptosis occurs by two different pathways. First, canonical pyroptosis is supported by the caspase (CASP)-1 activation by inflammasomes, which recognize pathogen-associated molecular patterns (PAMPs) and damage-associated molecular patterns (DAMPs)(red arrows). Second, the noncanonical pyroptosis is conducted by the activation of caspase-1 and caspase-4/-5 (caspase 11 in mice), which can be directly triggered by lipopolysaccharide (LPS) independent of TLR4 (Toll-like receptor 4)(purple arrows). Gasdermin D N-terminal fragments, generated by activated caspases, oligomerize and create pores in the plasma membrane due to their binding to certain lipids in the plasma membrane inner leaflet, leading to the release of cellular contents and cell death (violet arrows). Caspase-4/-5/-11, on the other hand, activates the Pannexin-1 channel and opens the P2X_7_ pore to induce pyroptosis. Activated Pannexin-1 can trigger the NLRP3 inflammasome through K^+^ efflux, and caspase-1 is activated by cleaved Gasdermin D through the combination of NLRP3 and ASC (dashed arrows). Caspase-1 activation results in the proteolytic cleavage of pro-IL-1β and pro-IL-18 and the formation of mature IL-1β and IL-18 [41].

**Figure 3:**
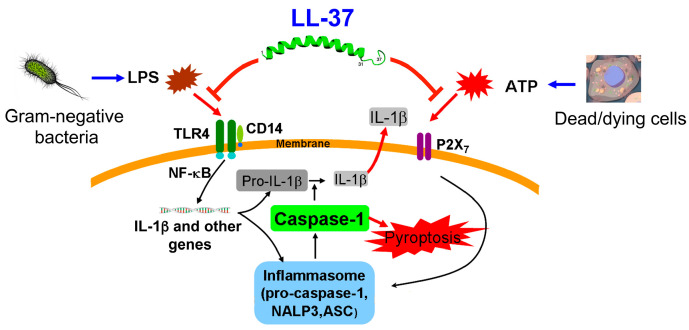
Suppression of LPS/ATP-induced macrophage pyroptosis by LL-37. Gram-negative bacterial LPS and dead/dying cell-derived ATP induce macrophage pyroptosis via the action on CD14/TLR4 and P2X_7_, respectively. LL-37 reduces the LPS/ATP-stimulated pyroptosis of macrophages and IL-1β production by both curtailing the action of LPS on CD14/TLR4 and blocking the P2X_7_ response to ATP.

Pyroptosis is a cell death pathway that is dependent on caspase-1 and occurs mostly in dendritic cells and macrophages, in association with the release of pro-inflammatory cytokines (IL-1β and IL-18) [7] (Figure 2). In addition, membrane perforation leads to cytosolic content release, which augments the inflammatory reactions [7,35]. Two separate stimuli, microbial-pathogen-associated molecular patterns (PAMPs) (including bacterial lipoproteins and LPS) and endogenous damage-associated molecular patterns (DAMPs) (for example, ATP and uric acid) are required for inducing pyroptosis [8,36]. These two stimuli trigger the formation of an inflammasome, a multi-protein complex (typically including caspase-1, NALP3 (NACHT domain-, leucine-rich repeat-, and pyrin domain (PYD)-containing protein 3) and ASC (apoptosis-associated speck-like protein containing a CARD)), which facilitates the conversion of pro-caspase-1 to active caspase-1 [37]. Subsequently, the activated caspase-1 catalyzes the cleavage of pro-IL-1β and pro-IL-18 to release IL-1β and IL-18, respectively, and N-terminal fragments of Gasdermin D, generated by activated caspase-1, oligomerize and create pores in the plasma membrane, leading to cell death (pyroptosis) [8,37]. It is important to note that caspase-1 genetic deletion leads to suppressed IL-1β levels and confers a protective effect on murine sepsis models and improves the survival of mice [38,39]. Therefore, the activation of caspase-1 and the resultant pyroptosis plays a significant part in sepsis pathogenesis and mortality [7,35]. Recently, we described that the LPS/ATP-induced pyroptosis as well as production of IL-1β are suppressed by LL-37 in macrophages by both curtailing the effect of LPS on CD14/TLR4 (Toll-like receptor 4) and preventing the P2X_7_ response to ATP in vitro [40] (Figure 3). Accordingly, we hypothesize that LL-37 exhibits protective effects in the murine septic model by inhibiting the pyroptosis of macrophages in vivo [32].

**Figure 4:**
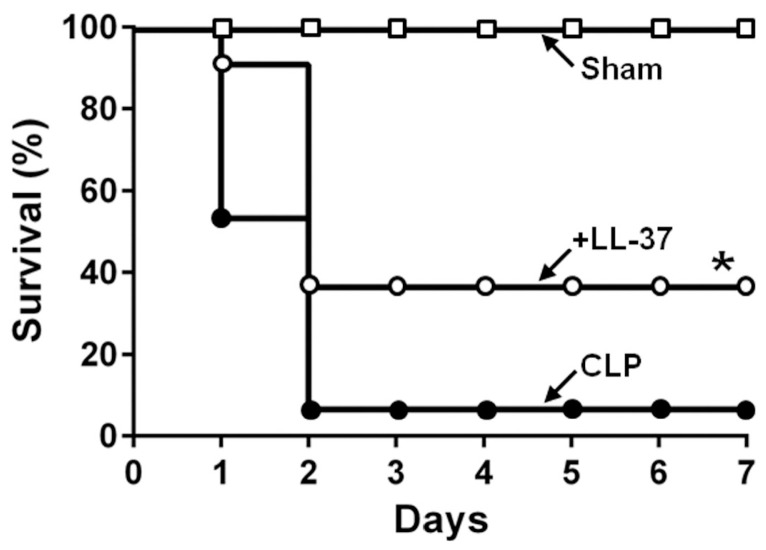
Effect of LL-37 on the survival of cecal ligation and puncture (CLP) septic mice. Mice were divided into the Sham (without CLP; □), CLP (●) and LL-37 (○) groups. In the LL-37 group, mice were intravenously administered with 2 μg per mouse LL-37 immedeiately after CLP, and the survival rates of the mice were monitored for 7 days. Survival data were analyzed using the Kaplan-Meier method and survival curves were compared using the log-rank test and Gehan-Breslow-Wilcoxon test in univariate analysis. *n* = 8–15 per group. * *p* < 0.05 [32].

**Figure 5:**
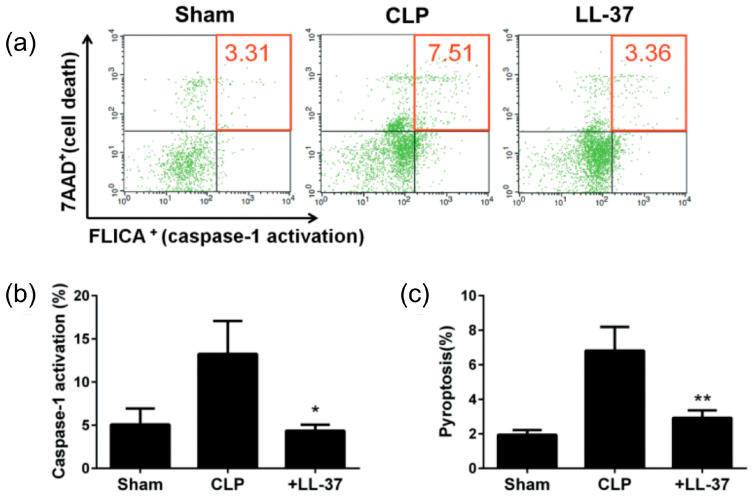
Effect of LL-37 administration on the pyroptosis of peritoneal macrophages in CLP mice. Peritoneal cells were collected from mice of Sham, CLP and LL-37 groups at 5 h after the surgery. Thereafter, peritoneal cells were evaluated for pyroptosis by detecting caspase-1 activation (FLICA positive) and cell death (7AAD positive) of the peritoneal macrophages (F4/80 positive) by flow cytometry. In panel (**a**), upper halves, right halves and upper-right quadrants show cell death, caspase-1 activation and pyroptosis (FLICA/7AAD-double positive), respectively, among peritoneal macrophages. Panels (**b**) and (**c**) show the percentage of caspase-1 activation and pyroptosis, respectively. Values are compared between the CLP and LL-37 groups. * *p* < 0.05, ** *p* < 0.01. FLICA, fluorescent-labeled inhibitor of caspases; 7AAD, 7-amino-actinomycin D [32].

**Figure 6:**
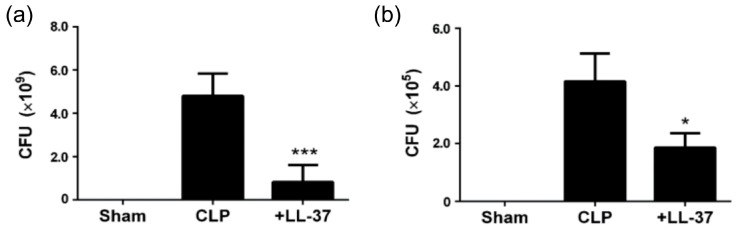
Effect of LL-37 administration on the bacterial burdens in peritoneal fluids and blood samples of CLP mice. Peritoneal fluids and blood samples were collected from mice of Sham, CLP and LL-37 groups at 15 h after the surgery, and serially diluted in PBS. Then, diluted samples were plated on Trypto-Soya agar plates, and the plates were incubated for 20 h at 37 °C. CFU was counted and corrected for the dilution factor. Panels (**a**) and (**b**) show the CFU in the peritoneal fluids and blood samples, respectively. Values are compared between the CLP and LL-37 groups. * *p* < 0.05, *** *p* < 0.001 [32].

The results indicated that the administration of LL-37 (2 μg/mouse) intravenously, to the CLP septic mice, improves their survival (Figure 4), and the effect was dose-dependent, since LL-37 (1 μg/mouse and 2 μg/mouse) improved the survival rate to 14.3% and 36.4%, respectively [32]. Interestingly, the pyroptosis of peritoneal macrophages and the CLP-induced caspase-1 activation are inhibited by LL-37 (Figure 5). In addition, the levels of inflammatory cytokines (IL-1β, IL-6 and TNF-α) in both the peritoneal fluids and sera were reduced by LL-37, which also blocked the peritoneal macrophages activation (as assessed by the elevated intracellular levels of IL-1β, IL-6 and TNF-α). Finally, LL-37 decreased the bacterial load in both the peritoneal fluids as well as blood samples (Figure 6). Thus, our work indicates that LL-37 administration improves the survival of CLP septic mice by inhibiting activation and pyroptosis of macrophages, production of inflammatory cytokines, and bacterial growth.

However, the mechanism by which LL-37 prevents pyroptosis and the caspase-1 activation in the CLP model, in vivo, is not known. In addition, LL-37, due to its antimicrobial activity, may protect the CLP septic mice by lowering the burden of bacteria in the blood and by decreasing the pyroptosis of macrophages.

Furthermore, it has been reported that LPS, a major component of bacteria, is elevated in the sera and peritoneal fluids of sepsis models [42]. Moreover, dead/dying cells release ATP extracellularly, and thus it is increased in the plasma of CLP mice [43] and peritoneal fluid of *E. coli*-induced septic mice [44]. Importantly, we earlier described that LPS and ATP together induce macrophage pyroptosis, which is blocked by LL-37 through both LPS neutralization and inhibition of the P2X_7_ activation by ATP [40]. Thus, we propose that in the CLP septic mice, LPS and ATP are the primary inducers of caspase-1 activation and pyroptosis, as in the case of other sepsis models [45,46], and that LL-37 prevents the actions of LPS and ATP, both in vivo and in vitro, thereby inhibiting pyroptosis (caspase-1 activation) and improving the survival of CLP mice (Figure 3).

## 3. LL-37 induces NET Release from Neutrophils and Reduces the Inflammatory Response in CLP Mice

**Figure 7:**
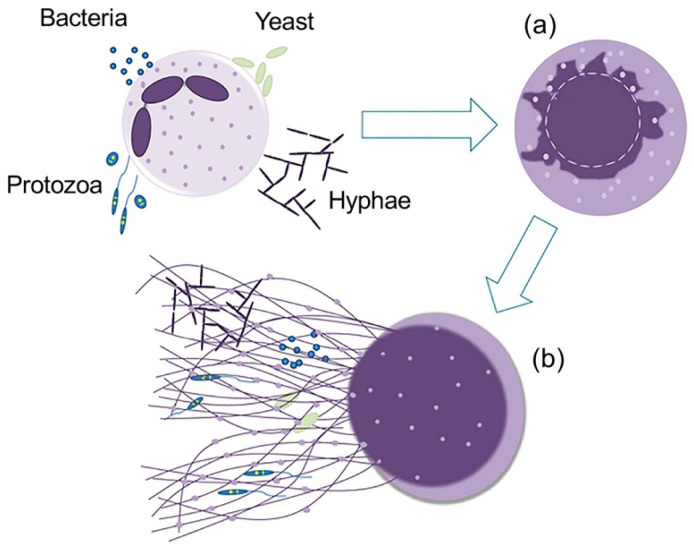
Mechanism for the release of neutrophil extracellular traps (NETs). Bacteria, protozoa, different fungi (yeast and hyphae forms) or their products cause the stimulation of neutrophils, leading to: (**a**) ultrastructural changes in the shape of nuclei by the decondensation of chromatin, nuclear membrane swelling and fragmentation, facilitate the chromatin association with granules and cytoplasmic proteins, and (**b**) release of extracellular structures that contain histones decorated with DNA, granular and cytoplasmic proteins of neutrophils, which are capable of trapping and killing the microorganisms [47].

Neutrophils function as the first line of host defense against invading microorganisms [11,12]; and they exert antimicrobial activity through phagocytosis and the subsequent killing of microorganisms by the actions of reactive oxygen species and antimicrobial granule proteins or peptides. Moreover, upon stimulation, neutrophils undergo NETosis and release NETs [13] (Figure 7). NETs are capable of trapping the microorganisms and by the action of NET-associated components (DNA, histone and granule proteins and peptides) show anti-bacterial activity [14,15].

As described above, the intravenous administration of LL-37 enhanced the survival of murine CLP sepsis model [32]. Moreover, it is demonstrated that LL-37 induces NET release from neutrophils in vitro [48]. Thus, we hypothesized that LL-37 may induce the release of NETs from neutrophils in vivo and protect mice from CLP-induced sepsis. To test this hypothesis, we evaluated the effect of LL-37 on the levels of inflammatory cytokines (TNF-α and IL-1β), triggering receptor expressed on myeloid cells (TREM)-1 and DAMPs (histone-DNA complex and high-mobility group protein 1 (HMGB1)) as well as NETs (determined as myeloperoxidase (MPO)-DNA complex) in plasma and peritoneal fluids of CLP septic mice [33].

**Figure 8:**
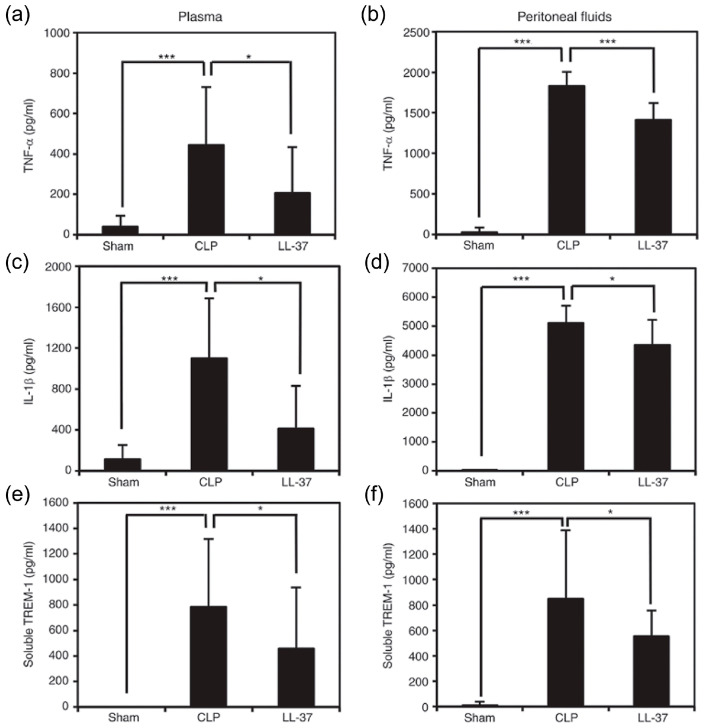
Effect of LL-37 administration on the levels of TNF-α, IL-1β and soluble TREM-1 in plasma and peritoneal fluids of CLP mice. Plasma and peritoneal fluids were collected from mice of Sham, CLP and LL-37 groups at 20 h after the surgery, and assayed for TNF-α, IL-1β and soluble TREM-1 by ELISA. TNF-α levels (**a**) and (**b**), IL-1β levels (**c**) and (**d**), and TREM-1 levels (**e**) and (**f**) in plasma and peritoneal fluids, respectively. Values are compared among Sham, CLP and LL-37 groups.* *p* < 0.05, *** *p* < 0.001 [33].

**Figure 9:**
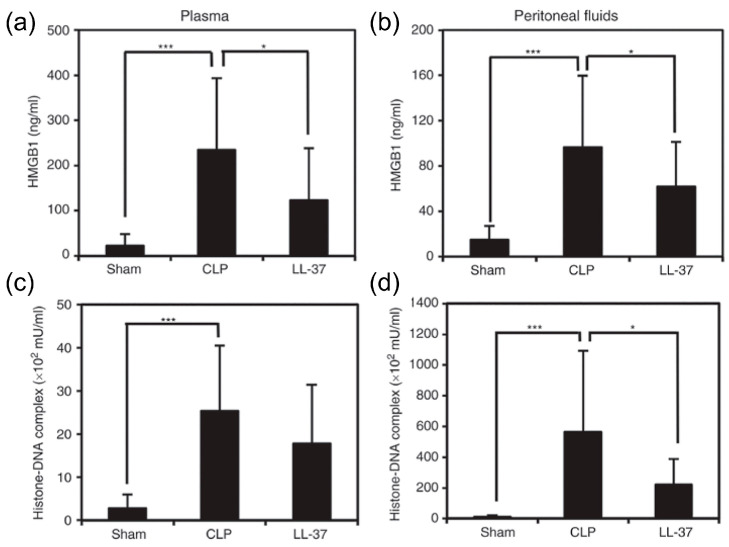
Effect of LL-37 administration on the levels of HMGB1 and histone-DNA complex in plasma and peritoneal fluids of CLP mice. Plasma and peritoneal fluids were collected from mice of Sham, CLP and LL-37 groups at 20 h after the surgery, and assayed for HMGB1 and histone-DNA complex by ELISA. HMGB1 levels (**a**) and (**b**), and histone-DNA complex levels (**c**) and (**d**) in plasma and peritoneal fluids, respectively. Values are compared among Sham, CLP and LL-37 groups. * *p* < 0.05, *** *p* < 0.001 [33].

**Figure 10:**
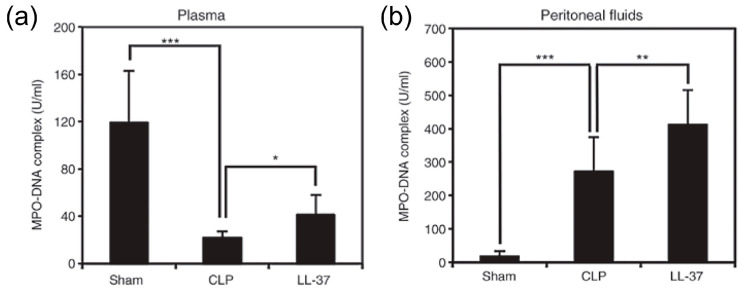
Effect of LL-37 administration on the levels of MPO-DNA complex in plasma and peritoneal fluids of CLP mice. Plasma and peritoneal fluids were collected from mice of Sham, CLP and LL-37 groups at 20 h after the surgery, and NETs were assayed as MPO-DNA complex by ELISA. Levels of MPO-DNA complex (**a**) and (**b**) in plasma and peritoneal fluids, respectively. Values are compared among Sham, CLP and LL-37 groups. * *p* < 0.05, ** *p* < 0.05, *** *p* < 0.001 [33].

**Figure 11:**
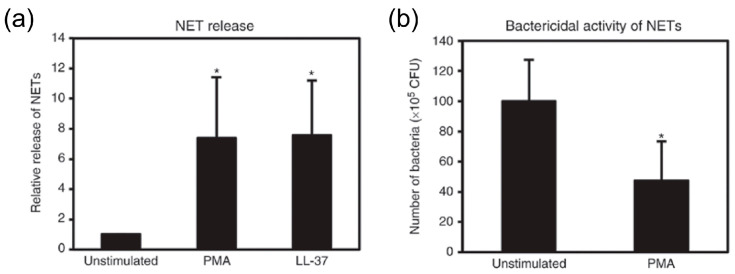
Induction of NET release and bactericidal activity of NETs. Mouse bone marrow-derived neutrophils (10^6^ cells) were incubated without (unstimulated) or with 100 nM PMA (phorbol myristate acetate) or 5 μM LL-37 for 4 h at 37 °C. Then, the supernatants were recovered, and assayed for MPO-DNA complex by ELISA (**a**). Relative NET release from PMA- or LL-37-stimulated neutrophils is expressed as a ratio to that from unstimulated neutrophils. Bactericidal activity of NETs was assayed by incubating the supernatants from unstimulated or PMA-stimulated neutrophils with *E. coli* (10^7^ cells) in LB broth at room temperature for 30 min (**b**). Mixtures were appropriately diluted, and plated on Trypto-Soya agar plates. The plates were incubated for 20 h at 37 °C, and CFU were counted and corrected for the dilution factor. Values are compared between unstimulated and PMA-or LL-37-stimulated neutrophils. * *p* < 0.05 [33].

The findings suggested that LL-37 administration, intravenously, prevented the upsurge in DAMPs as well as TNF-α, IL-1β and soluble TREM-1 in peritoneal fluids and plasma (Figure 8 and Figure 9). Interestingly, LL-37 significantly reduced the increase in the number of peritoneal polymorphonuclear cells (neutrophil) during sepsis. In addition, LL-37 lowered the microbial burden in circulation and also in peritoneal fluids. Importantly, LL-37 administration significantly increased the level of NETs in plasma and peritoneal fluids of CLP mice (Figure 10). In addition, we established in vitro that LL-37 directly stimulates bone marrow-derived neutrophils to release NETs and the NETs possess the bactericidal activity (Figure 11).

TREM-1 is expressed mainly on monocytes/macrophages and neutrophils and is recognized as a novel receptor, which participates in the amplification of inflammatory responses in sepsis [49]. Moreover, membrane-anchored TREM-1 is shed by metalloproteinases as a soluble form of TREM-1 [50], and soluble TREM-1 can be used as a potential marker for identifying clinically ill patients with infection [51].

DAMPs are host cell-derived biomolecules that function as potent activators of innate immune system initiating systemic inflammatory response syndrome (SIRS), multiple organ failure and death in sepsis [52]. DAMPs consist of nuclear or cytosolic molecules, such as DNA, histone, HMGB1 and ATP, and are released outside the cells following tissue injury or cell death [8,53].

Based on these findings, our observations suggest that LL-37 stimulates the release, in vivo, of bactericidal NETs, thereby suppressing the bacterial growth and improving the inflammatory responses of sepsis, as evidenced by the suppression of inflammatory cytokines, soluble TREM-1 and DAMPs (host cell death) and the change of inflammatory cell number, and protects mice from lethal sepsis [33].

## 4. LL-37 Stimulates the Release of Antimicrobial Ectosomes from Neutrophils and Improves the Septic Condition

**Figure 12:**
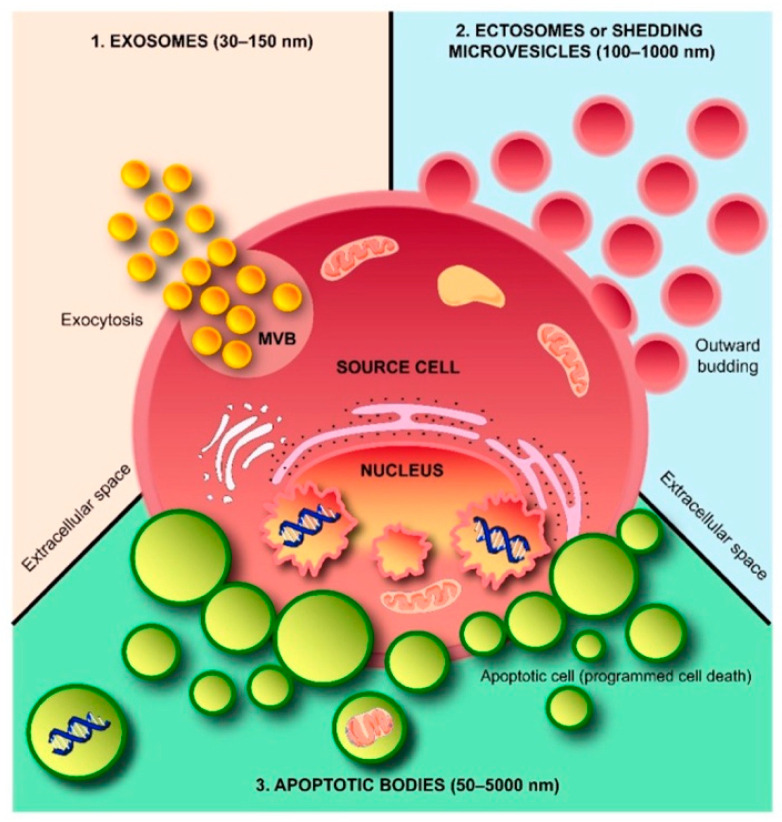
Schematic representation of extracellular vesicles released from cells. Three subtypes of extracellular vesicles are secreted by cells, and these are exosomes, ectosomes (shedding microvesicles) and apoptotic bodies. Exosomes arise via exocytosis, whereas plasma membrane outward budding generates ectosomes. On the other hand, apoptotic bodies are produced by cells undergoing later stages of apoptosis. MVB: multivesicular body [17].

Various host cells release extracellular vesicles including ectosomes (100-1000 nm in diameter) and exosomes (30–150 nm in diameter) that mediate intercellular communications [16,17] (Figure 12). During the inflammatory response, neutrophils release microvesicles, named ectosomes, which bud off from the cell membrane [16,17]. Ectosomes express the cell surface molecules originating from neutrophils such as Ly6G and phosphatidylserine, and contain functional proteins of neutrophils [17,18]. Interestingly, ectosomes released from bacteria-stimulated neutrophils exhibit bacteriostatic potential [19]. Moreover, the ectosome level is reported to be augmented in surviving patients of sepsis [20,21]; thus, ectosomes are speculated to play a protective role in sepsis. Since the survival of a murine CLP sepsis model is improved by LL-37 [32,33], we investigated the mechanisms of LL-37-mediated protective action against sepsis, by addressing the effect of LL-37 on the release of ectosomes in the CLP model [34].

**Figure 13:**
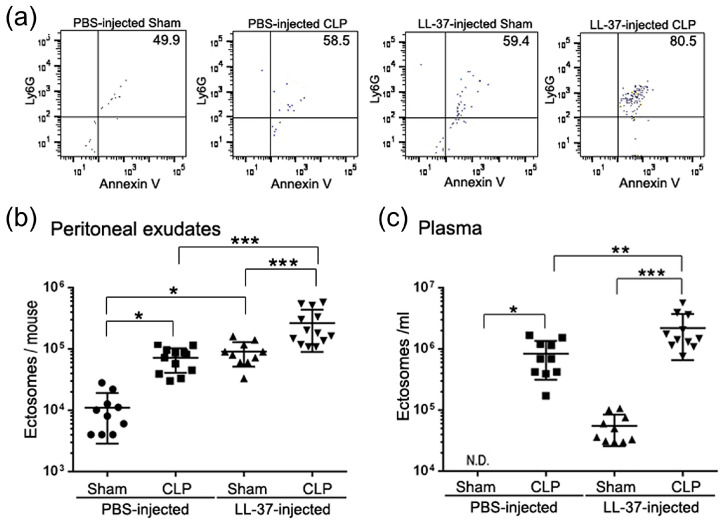
Effect of LL-37 administration on the ectosome level in peritoneal exudates and plasma of CLP mice. Peritoneal exudates and plasma were collected from mice of Sham and CLP groups with PBS- or LL-37-injection at 14–16 h after the surgery, microvesicles isolated from peritoneal exudates and plasma were incubated with PE-anti-Ly6G IgG and FITC-Annexin V, and analyzed by flow cytometry. Ectosomes express the cell surface molecules originated from neutrophils such as Ly6G (a neutrophil surface marker) and phosphatidylserine, and defined as double-positive (Ly6G^+^/Annexin V^+^ particles) for both anti-Ly6G antibody and Annexin V (a substance with an ability to bind to phosphatidylserine). (**a**) Representative cytograms of microvesicles from peritoneal exudates of PBS- or LL-37-injected Sham or CLP mice are shown. The counts of ectosomes in peritoneal exudates (**b**) and plasma (**c**) are shown. Values were compared between PBS-injected Sham and CLP mice, LL-37-injected Sham and CLP mice, PBS-injected and LL-37-injected Sham mice, and PBS-injected and LL-37-injected CLP mice. * *p* < 0.05, ** *p* < 0.01, *** *p* < 0.001. N.D. (not detected); ectosomes could not be counted in the plasma of PBS-injected Sham mice [34].

**Figure 14:**
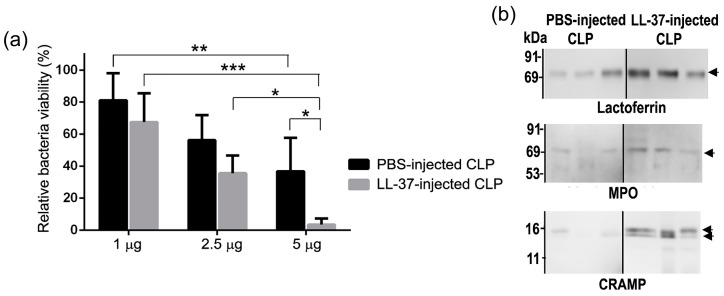
Antibacterial activity and detection of antibacterial molecules in ectosome fractions isolated from peritoneal exudates of CLP mice. (**a**) Ectosome fractions (1, 2.5 or 5 μg protein) isolated from peritoneal exudates of PBS-injected (PBS-injected CLP) or LL-37-injected CLP (LL-37-injected CLP) mice were incubated with *E. coli* for 20 min at 37 °C. The relative bacteria viability is calculated and expressed as the percentage by dividing the colony-forming unit (cfu) of bacteria incubated with ectosome fractions by that with vehicle (PBS). Values were compared between ectosome fractions from PBS-injected CLP and LL-37-injected CLP mice, and among ectosome fractions (containing 1, 2.5 and 5 μg protein) isolated from PBS-injected CLP or LL-37-injected CLP mice. * *p* < 0.05, ** *p* < 0.01, *** *p* < 0.001. (**b**) Ectosome fractions (1 μg protein) isolated from peritoneal exudates of PBS-injected CLP and LL-37-injected CLP mice were subjected to SDS-PAGE, followed by western blotting using anti-lactoferrin, MPO, and CRAMP antibodies. Each lane shows an ectosome fraction isolated from each of PBS-injected CLP and LL-37-injected CLP mouse [34].

The findings showed that administration of LL-37 enhances the level of ectosomes in the peritoneal exudates and plasma of CLP-operated mice as well as Sham (the same procedure but without ligation and puncture) mice (Figure 13), suggesting that the enhanced level of ectosomes may be associated with the survival of CLP mice. In addition, the bacterial load was decreased in LL-37-injected CLP mice compared with PBS-injected CLP mice; thus, it could be speculated that the enhanced level of ectosomes is associated with the lower bacterial load in LL-37-injected CLP mice. To further confirm the antibacterial activity of ectosomes, we assessed the antibacterial activity of ectosome fractions isolated from PBS- and LL-37-injected CLP mice. Importantly, both fractions possessed the antibacterial potential, and interestingly, the fraction from LL-37-injected CLP mice displayed higher potential than that from PBS-injected CLP mice (Figure 14a). These observations suggest that LL-37 elevates the level of ectosomes with higher antibacterial potential, thereby reducing the bacterial load in CLP mice.

Since neutrophil granule molecules are contained in ectosomes [17,18], we examined the involvement of neutrophil granule molecules in the antibacterial activity of ectosomes. Western blot analysis indicated that the neutrophil granule-derived antimicrobial molecules, such as lactoferrin, MPO and CRAMP (cathelicidin-related antimicrobial peptide, a mouse ortholog of human cathelicidin peptide) were detected in the ectosome fractions isolated from peritoneal exudates of both PBS- and LL-37-injected CLP mice. In addition, the fraction from LL-37-injected CLP mice had increased amounts of these antimicrobial molecules compared with that from PBS-injected CLP mice (Figure 14b). In addition, anti-lactoferrin or anti-CRAMP antibody partially but substantially abrogated the antibacterial activity of ectosome fractions from PBS- and LL-37-injected CLP mice, suggesting the involvement of lactoferrin and CRAMP in the antibacterial potential of ectosomes. Collectively, these findings indicate that LL-37 stimulates the release of ectosomes containing higher amounts of antibacterial molecules (lactoferrin and CRAMP) in CLP mice, which may exert the antibacterial action and reduce the bacterial load, thereby improving the survival of septic mice.

**Figure 15:**
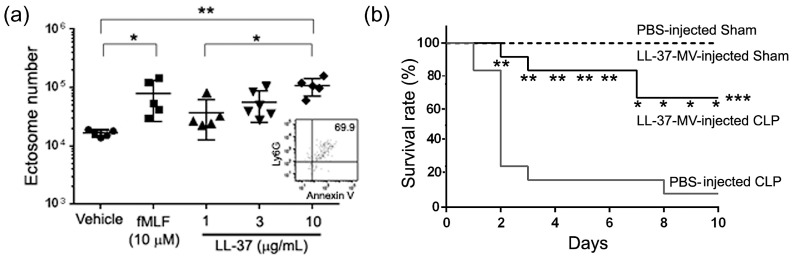
Effect of LL-37 on the release of ectosomes from mouse bone marrow-derived neutrophils, and effect of ectosome administration on the survival of CLP mice. (**a**) Mouse bone marrow-derived neutrophils (2 × 10^6^ cells) were stimulated with fMLF or LL-37 for 45 min at 37 °C, and the released ectosomes in the supernatant were analyzed by flow cytometry. Values were compared between the incubation with vehicle (PBS) and fMLF or LL-37, and among 1, 3 and 10 μg/mL of LL-37. An inlet shows a representative cytogram of ectosomes released from LL-37 (10 μg/mL)-stimulated neutrophils. (**b**) CLP mice were intraperitoneally injected with PBS (PBS-injected CLP) or ectosomes (3 × 10^5^ ectosomes) isolated from LL-37-stimulated neutrophils (LL-37-MV-injected CLP) 2 h after the surgery. Sham mice were also injected with PBS (PBS-injected Sham) or ectosomes (LL-37-MV-injected Sham) 2 h after the surgery. The survival was monitored for 10 days, and the survival rate was compared between PBS-injected CLP and LL-37-MV-injected CLP mice by Kaplan-Meier method for 10 days and by chi square analysis for each day. * *p* < 0.05, ** *p* < 0.01,*** *p* < 0.001 [34].

Moreover, we examined whether LL-37 is able to stimulate neutrophils ex vivo, to release ectosomes and whether the administration of ectosomes ameliorates a murine septic CLP model in vivo. Importantly, LL-37 directly activated mouse bone marrow-derived neutrophils, ex vivo, to release ectosomes (Figure 15a). In addition, the LL-37-triggered ectosomes have the antibacterial potential, and the administration of these ectosomes to CLP mice led to improved survival of mice and reduced bacterial load (Figure 15b).

Collectively, these findings indicate that LL-37 stimulates neutrophils to release antibacterial ectosomes, thereby eliminating bacteria, and protecting mice from lethal sepsis.

## 5. Perspective

Cationic antimicrobial peptides target cell surface anionic lipids such as phosphatidyl glycerol and cardiolipin that are abundant in microorganisms; the action is not receptor-based but involves a less specific interaction with microbial membrane components [54,55]. In contrast, the mammalian cell membrane is mainly composed of electrically neutral phospholipids such as phosphatidylcholine and sphingomyelin, for which the affinity of the antimicrobial peptides is generally low [53]. The simple electrostatic interaction between cationic antimicrobial peptides and microbial membrane lipids provides selective toxicity (bacteria versus mammalian cells) as well as a broad spectrum of antimicrobial activities. Moreover, the development of microbial resistance is assumed to be low, because the target molecules (anionic lipids) are important components conserved among microorganisms, and the molecular recognition between cationic peptides and target molecules is rather lenient [54,55]. In addition, the peptides are small and relatively easy to synthesize. From these points of view, cationic antimicrobial peptides could be promising candidates for new antibiotics with therapeutic value. Moreover, cationic host defense peptides are expected to protect the host against pathogens not only by being directly antimicrobial but also by modulating the immune responses and boosting infection-resolving immunity while dampening potentially harmful pro-inflammatory (septic) responses [56,57,58,59].

However, it should be noted that as cationic antimicrobial peptides act principally via electrostatic attraction, and hydrophobic partitioning into the membrane targets, they could also bind to various host components such as anionic constituents of host cell membranes, leading to potentially harmful side effects on the host [55]. In this context, it has been demonstrated that high concentrations of cationic antimicrobial peptides are occasionally toxic to host cells [26]. Thus, cationic antimicrobial peptides should be cautiously administered in vivo, considering their toxic effects on host cells.

**Figure 16:**
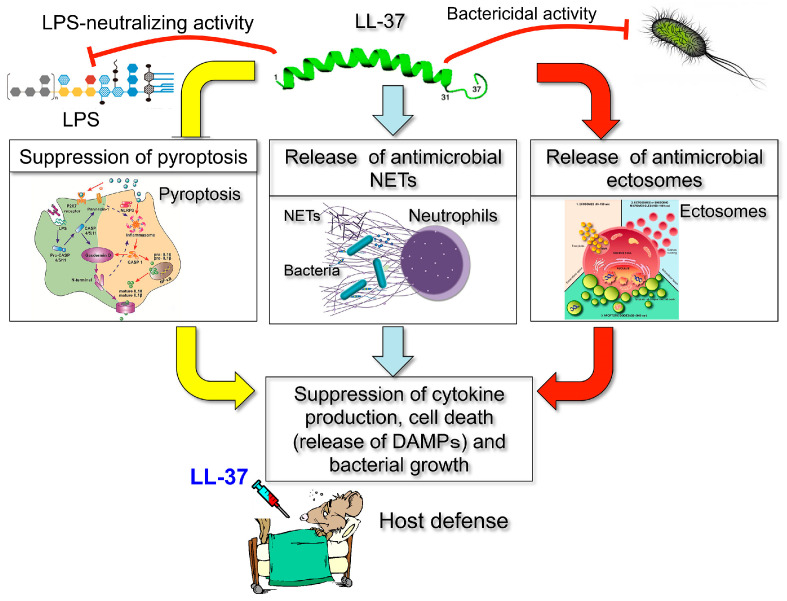
Therapeutic action of antimicrobial cathelicidin peptide LL-37 on a murine sepsis model. LL-37 protects CLP septic mice through at least three mechanisms, i.e., the suppression of pro-inflammatory macrophage pyroptosis (yellow arrows) and the release of antimicrobial NETs (blue arrows) and ectosomes (red arrows) from neutrophils, in addition to its own bactericidal and LPS-neutralizing activities.

Our studies have revealed that LL-37 exhibits multiple functions in sepsis. Firstly, LL-37 enhances the survival of CLP mice by reducing the macrophage pyroptosis that induces the secretion of pro-inflammatory cytokines (such as IL-1β) and augments inflammatory reactions in sepsis [32]. Secondly, LL-37 induces the release of NETs with potent bactericidal activity and protects mice from CLP-induced sepsis [33]. Thirdly, LL-37 stimulates neutrophils to release antimicrobial ectosomes, which improve murine sepsis [34]. These findings indicate that LL-37 protects CLP septic mice through at least three mechanisms, i.e., the suppression of pro-inflammatory macrophage pyroptosis and the release of antimicrobial NETs (induction of NETosis) and ectosomes from neutrophils. Thus, LL-37 can be a promising candidate as a therapeutic agent for sepsis because of its multiple functions, including modulation of cell death (pyroptosis and NETosis) and release of antimicrobial NETs and ectosomes as well as its own bactericidal and LPS-neutralizing activities (Figure 16). Furthermore, our findings likely open novel paths for designing immunomodulatory peptide drugs, using LL-37 as the lead molecule with multiple actions on the pathogenesis of sepsis.
